# Enhancing Breast Density Assessment in Mammograms Through Artificial Intelligence

**DOI:** 10.1007/s10278-025-01657-6

**Published:** 2025-09-05

**Authors:** Naila Camila da Rocha, Abner Macola Pacheco Barbosa, Yaron Oliveira Schnr, Lucas Dias Borges Peres, Luis Gustavo Modelli de Andrade, Guilherme Jordao de Magalhaes Rosa, Eduardo Carvalho Pessoa, Jose Eduardo Corrente, Liciana Vaz de Arruda Silveira

**Affiliations:** 1 University of Wisconsin-Madison, 1675 Observatory Dr, Madison, WI 53706 USA; 2 Sao Paulo State University (UNESP), Rubião Jr, Botucatu, São Paulo, SP 18618-687 Brazil

**Keywords:** Convolutional neural networks, Breast density, Mammography, Breast cancer, Computer-aided diagnosis

## Abstract

Breast cancer is the leading cause of cancer-related deaths among women worldwide. Early detection through mammography significantly improves outcomes, with breast density acting as both a risk factor and a key interpretive feature. Although the Breast Imaging Reporting and Data System (BI-RADS) provides standardized density categories, assessments are often subjective and variable. While automated tools exist, most are proprietary and resource-intensive, limiting their use in underserved settings. There is a critical need for accessible, low-cost AI solutions that provide consistent breast density classification. This study aims to develop and evaluate an open-source, computer vision-based approach using deep learning techniques for objective breast density assessment in mammography images, with a focus on accessibility, consistency, and applicability in resource-limited healthcare environments. Our approach integrates a custom-designed convolutional neural network (CD-CNN) with an extreme learning machine (ELM) layer for image-based breast density classification. The retrospective dataset includes 10,371 full-field digital mammography images, previously categorized by radiologists into one of four BI-RADS breast density categories (A–D). The proposed model achieved a testing accuracy of 95.4%, with a specificity of 98.0% and a sensitivity of 92.5%. Agreement between the automated breast density classification and the specialists’ consensus was strong, with a weighted kappa of 0.90 (95% CI: 0.82–0.98). On the external and independent mini-MIAS dataset, the model achieved an accuracy of 73.9%, a precision of 81.1%, a specificity of 87.3%, and a sensitivity of 75.1%, which is comparable to the performance reported in previous studies using this dataset. The proposed approach advances breast density assessment in mammograms, enhancing accuracy and consistency to support early breast cancer detection.

**Supplementary Information:**

The online version contains supplementary material available at 10.1007/s10278-025-01657-6.

## Introduction

Breast cancer, or mammary carcinoma, is the most commonly diagnosed cancer and the leading cause of cancer-related deaths among women worldwide, with mortality rates rising significantly after the age of 40 [1, 2]. Infiltrating ductal carcinoma is the most prevalent histological type, accounting for approximately 80–90% of all breast cancer cases [3, 4]. Established risk factors include age, hormonal and reproductive history, environmental exposures, and notably, breast density [3–5]. High breast density is not only an independent risk factor for breast cancer but also hinders early detection by reducing the sensitivity of mammography [5].

Mammographic breast density refers to the proportion of fibroglandular (dense) tissue relative to fatty tissue, as visualized on a mammogram [6, 7]. To standardize its assessment, the American College of Radiology developed the Breast Imaging Reporting and Data System (BI-RADS), which classifies breast density into four categories: (A) almost entirely fatty, (B) scattered fibroglandular densities, (C) heterogeneously dense, and (D) extremely dense [7]. While the fourth edition of BI-RADS used quartile-based thresholds, the fifth edition removed specific percentage cutoffs in favor of a qualitative approach, placing greater reliance on radiologist interpretation [7].

However, subjective visual assessments of breast density are known to be variable, with substantial inter- and intra-observer inconsistencies reported in multiple studies [8, 9]. These limitations have driven the development of computer-aided diagnosis (CAD) systems and artificial intelligence (AI)-based methods aimed at providing more objective and reproducible evaluations. Recent research has demonstrated that AI algorithms, particularly those based on deep learning, can match or even surpass human performance in breast density classification [10–13]. For instance, commercial tools such as DensityAI (Densitas) and PowerLook Density Assessment (iCAD) have shown encouraging results, with reported accuracies between 76 and 89%, and weighted kappa statistics ranging from 0.537 to 0.744 [11]. However, these commercial systems are expensive and require high-performance computing infrastructure, which limits their adoption, particularly in public and low-resource healthcare environments.

Although deep-learning approaches have shown promising accuracy for BI-RADS classification, most commercial systems are proprietary, and many academic works do not make the code or pre-trained models publicly available, limiting reproducibility and real-world deployment, particularly in resource-constrained settings [14, 15]. A practical search conducted in 2025 on GitHub, the largest and most widely used public code repository, identified only a small number of open-source projects that provide both the source code and pre-trained models, and even these require additional steps before deployment and real-world use. Furthermore, the vast majority of publicly available tools are focused on breast cancer detection rather than breast density assessment, highlighting the lack of accessible, reproducible, and deployment-ready open-source solutions for this specific task.

A growing body of research has focused on the application of deep learning, particularly convolutional neural networks (CNNs), to mammographic breast density assessment. For example, a study [8] developed a CNN-based computer-aided diagnosis (CAD) system incorporating preprocessing, region of interest (ROI) selection, data augmentation, and classification, achieving 98.5% accuracy on the mini-MIAS dataset. In another investigation, researchers evaluated InceptionResNetV2, a CNN pre-trained on ImageNet, for BI-RADS density classification, reporting 99% accuracy for binary classification and 97% for four-class classification [13]. Additionally, an article [16] proposed a residual CNN and employed Grad-CAM to generate saliency maps, assessing model interpretability and robustness across varying class distributions using 1,662 labeled mammography exams.

Several other works have emphasized transfer learning. For instance, one approach [9] utilized InceptionV3, fine-tuned with softmax outputs for the four BI-RADS density categories, and demonstrated efficient retraining with limited data using stochastic gradient descent. Likewise, another study [12] applied AlexNet, DenseNet, and ShuffleNet architectures to classify mammographic images by combining density features with benign and malignant mass information, achieving test accuracies as high as 99.7% with DenseNet. Despite these promising outcomes, many models rely heavily on pre-trained architectures, demand substantial computational resources, or remain inaccessible due to proprietary constraints.

While these developments are significant, critical gaps persist. Most existing models are trained on relatively small or non-diverse datasets, often lack external validation, and are rarely optimized for deployment in low-resource settings. Furthermore, some studies [17, 18] concentrate on binary classification tasks (e.g., healthy vs. cancerous), limiting their relevance to BI-RADS-based density classification workflows.

To address these challenges, our study proposes a low-cost, open-source framework for automated breast density classification using deep learning. Specifically, we introduce a custom-designed convolutional neural network (CD-CNN) integrated with an extreme learning machine (ELM) classification layer to balance accuracy and computational efficiency. Our model was trained using radiologist-annotated BI-RADS categories from full-field digital mammograms and validated both internally and externally, achieving strong agreement with expert consensus (weighted kappa = 0.90). However, this study has several limitations that should be addressed in future work. First, the model relies on BI-RADS assessments made by interpretive radiologists in routine clinical practice, which may introduce inherent variability. In addition, generalizability may be constrained by two factors: the small sample size (30 cases) used in the external specialist validation dataset, and the use of only one external source, the mini-MIAS database, for validation, rather than a broader array of diverse datasets.

By prioritizing accessibility, reproducibility, and practical deployment, particularly in underserved healthcare settings, this work contributes a scalable and interpretable solution for consistent and efficient breast density assessment.

## Materials and Methods

### Patients

The retrospective mammography database used for the classification of breast density is a subset of data from a Brazilian tertiary hospital, evaluated by the Breast Radiology Department from 2014 to 2021. The dataset comprises 10,371 full-field digital mammography images from 2472 unique patients, with a mean patient age of 55.2 ± 8.9 years. Most patients have 4 images (2 craniocaudal—CC and 2 mediolateral oblique—MLO), while some have only 2 images (1 CC and 1 MLO) due to previous mastectomy. This study was approved by the Research Ethics Committee on November 21, 2022 (protocol 5.766.599; CAAE 63415222.5.0000.5411) and registered in SIPAT (292/2022).

**Fig. 1:**
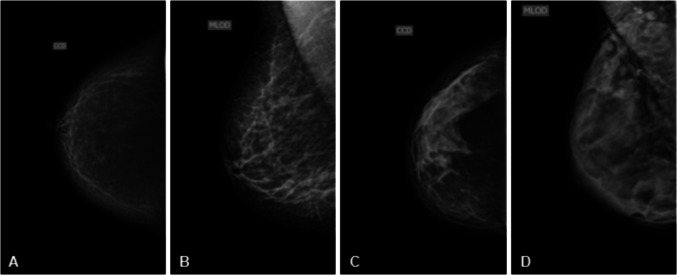
Sample images from dataset of the four breast density categories by BI-RADS

All images included in this study were independently assessed by at least two highly experienced breast imaging radiologists from the hospital’s Breast Radiology Department. These specialists are board-certified, have a minimum of five years of experience as specialists, and are members of both the Brazilian Society of Mastology and the National Committee for Breast Imaging of the Brazilian Federation of Gynecology and Obstetrics Associations (FEBRASGO). The double-reading was performed as part of the institution’s standard of care. Breast density was classified according to the BI-RADS breast density categories (A–D). Discrepancies were resolved by consensus discussion. Figure 1 shows representative examples from each BI-RADS density category.

Approximately 55% of the breasts were categorized as A or B (less dense), and 45% as C or D (dense). Only mammograms with acceptable positioning and a clearly stated breast density classification in the official radiology report were included. A summary of the dataset is presented in Table 1.

**Table 1:** Dataset description, including the total number of patients and the total number of mammography images, categorized by BI-RADS categories. Patient age is presented as the mean and standard deviation (SD)

**Name**	**Description**
Number of Images	10,371
Number of Patients	2,472
Mean patient age (years)	55.2 ± 8.9
A (Number of Images I Number of Patients)	1,052 I 253
B (Number of Images I Number of Patients)	4,626 I 1,105
C (Number of Images I Number of Patients)	4,190 I 996
D (Number of Images I Number of Patients)	503 I 118

To ensure robust model evaluation and prevent data leakage from repeated measures, a k-fold cross-validation strategy was employed. In each fold, all images from a given patient were assigned exclusively to either the training, validation, or test subset. This approach prevents the inclusion of correlated images from the same patient across different subsets, which could otherwise bias performance metrics.

### Model Selection and Architecture

A diverse set of models was evaluated, including custom-designed convolutional neural networks (CD-CNNs) and well-known pre-trained architectures from the Keras Applications library, such as VGG16 [19], VGG19 [19], ResNet50 [20], MobileNet [21], and DenseNet [22]. These pre-trained models were initialized with ImageNet weights, modified by removing the top classification layers (include_top = False), freezing the convolutional base layers, and adding custom fully connected layers for multi-class classification. This approach enabled transfer learning while adapting the models to the target task.

Additionally, three custom models (model 1, model 2, and model 3) were designed with varying numbers of convolutional, fully connected, and pooling layers to explore different network depths and complexities. model 1 consists of four convolutional layers, two fully connected layers, and four max-pooling layers. model 2 includes eight convolutional layers, two fully connected layers, and four max-pooling layers. model 3 has four convolutional layers, three fully connected layers, and four max-pooling layers, with an added dropout layer for regularization.

To ensure all models were trained and tested under the same conditions, standard parameters were established. Models were then fine-tuned by adjusting one parameter at a time, and the best model was selected based on common metrics, including testing accuracy, specificity, sensitivity, and computational training time (computational cost). The parameters tested included: resolution (50 × 50, 128 × 128, 224 × 224), number of iterations (100, 200, 300, 400, and 500), batch size (8, 16, 32, 64, and 128), hidden units ELM (64, 128, 256, and 512), and k-fold cross-validation (*k* = 5 or 10).

**Fig. 2:**
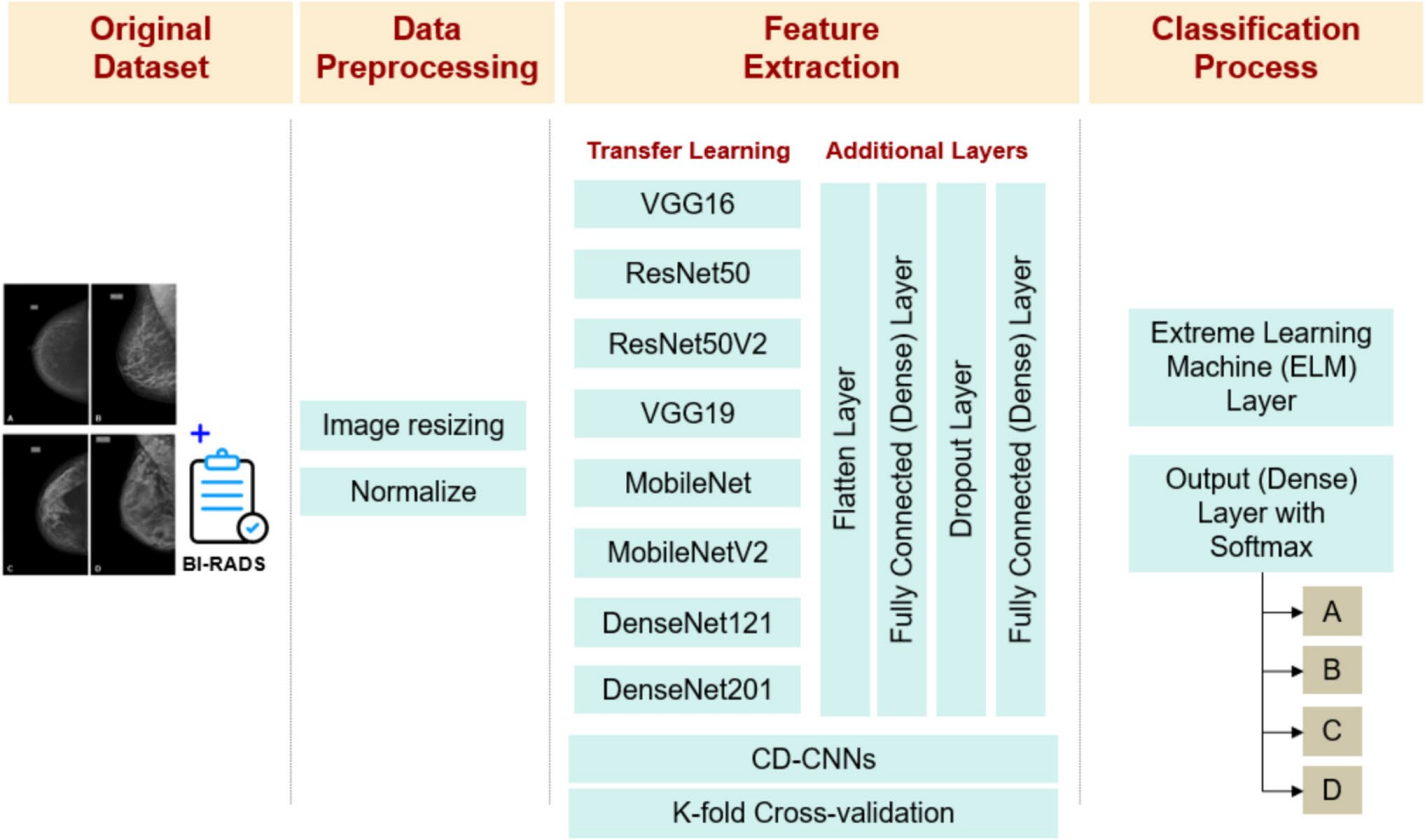
Diagram illustrating the implementation process of the model used in this study to classify breast density into the four BI-RADS categories. CD-CNN refers to the custom-designed convolutional neural network

For classifying breast density into the four BI-RADS categories, we employed categorical cross-entropy loss. The Adam optimizer was utilized to enhance accuracy, with a learning rate set to 0.0001 for all tests. All pre-trained models combined with extreme learning machine (ELM) included custom fully connected layers (CFCL) consisting of flattening, dense layers with ReLU activation, and a 50% dropout rate. The size of the last fully connected layer in the CNN was adjusted to match the number of hidden units in the ELM layer, ensuring consistent input dimensions, as illustrated in Fig. 2.

### Proposed System

**Fig. 3:**
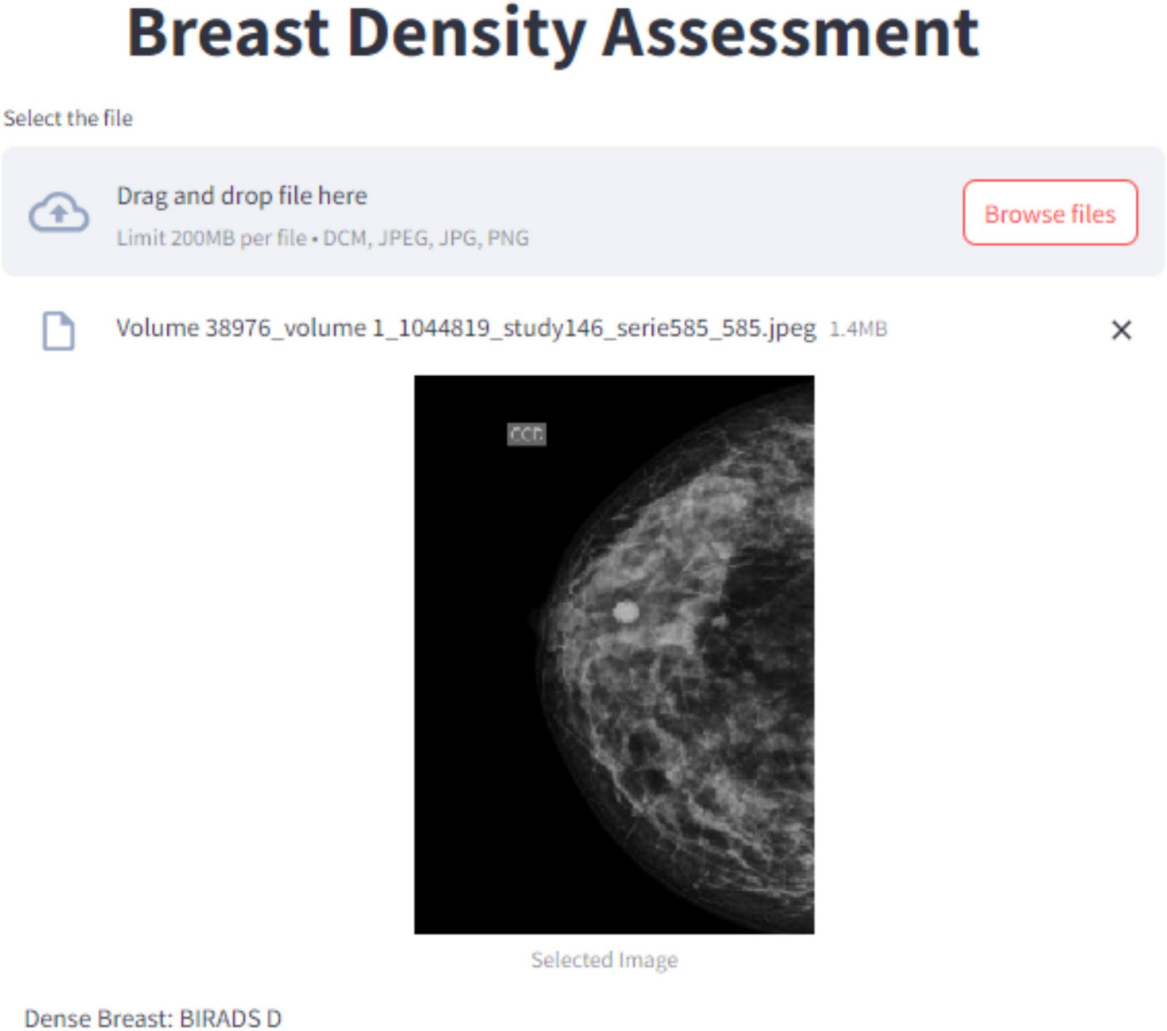
The app’s interface details, accessible at https://breastdensity.streamlit.app/

This research focuses on classifying breast density using mammographic images. The system categorizes input images into BI-RADS density categories A, B, C, or D based on features extracted from preprocessed mammograms. The system was developed, analyzed, and modeled using Python 3.12.3, with Streamlit utilized for deploying it as a web application. The source code is available on GitHub [23], and the proposed CAD system can be accessed directly at https://breastdensity.streamlit.app/. Details of the app’s interface are shown in Fig. 3.

An NVIDIA A100 GPU was used to accelerate the training of deep learning models. The A100 features 80 GB of high-bandwidth memory (HBM2e) and up to 312 teraflops of AI performance, making it ideal for handling the computationally intensive tasks associated with processing large mammographic datasets.

**Fig. 4:**
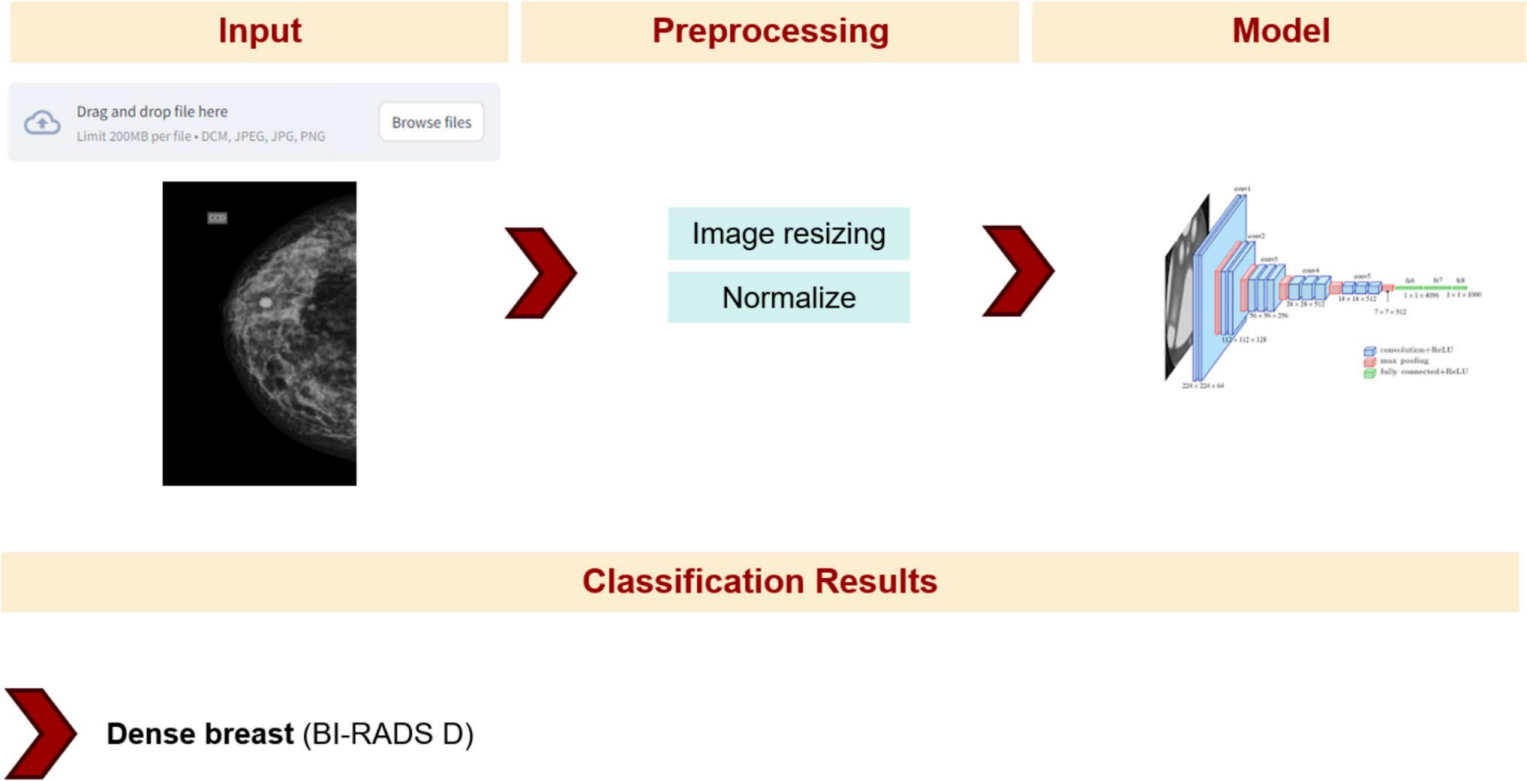
Workflow diagram illustrating the complete classification process for breast density using the proposed model

In the proposed workflow, users can upload images in DICOM, PNG, JPG, or JPEG format. Each image is preprocessed through resizing and normalization before being analyzed by the model, which then predicts the corresponding BI-RADS density category. Figure 4 illustrates the complete functioning of the proposed system.

### Specialist Validation

A prospective mammography dataset consisting of 30 randomly selected images was used for external validation. These images were not part of the system’s development phase. The dataset was submitted to the Breast Radiology Department for evaluation following the BI-RADS standard. To minimize classification bias, each mammogram was independently evaluated by two board-certified breast imaging specialists, both affiliated with the Brazilian Society of Mastology and the National Committee for Breast Imaging of FEBRASGO, each with at least five years of experience as a specialist.

To further ensure the reliability of the findings, inter-rater agreement between the specialists was calculated. Additionally, the consensus reached during a subsequent discussion was compared with the system’s density classification. This agreement was measured using the weighted kappa statistic, which provides a robust measure of inter-rater reliability and the degree of agreement beyond chance.

### External Validation

The Mammogram Image Analysis Society (MIAS) database comprises 322 images (161 pairs of left and right breast images). This open-source dataset was used as an external and independent dataset for validation, and the mammographic images are available via the Pilot European Image Processing Archive (PEIPA) at the University of Essex [24]. This database was chosen for its open-access availability and its long-standing role as a well-established resource for external validation in mammogram image analysis since 1994, although it does not include the same categories as the BI-RADS 5th Edition.

The MIAS database categorizes breast tissue density into three classes: fatty (F), fatty-glandular (G), and dense-glandular (D). To validate the system’s BI-RADS classification with these categories, the BI-RADS scores were mapped to the MIAS classes. If an image was classified as BI-RADS A or B and the MIAS category was fatty, it was identified as fatty (F). If the system classified the image as BI-RADS C or D and the MIAS category was Dense-glandular, it was identified as dense-glandular (D). For cases where the system’s classification was BI-RADS B or C and the MIAS category was fatty-glandular, it was interpreted as fatty-glandular (G), representing a transitional category between fatty and dense breasts. This approach enabled a clear distinction between dense, non-dense (fatty), and transitional breast tissue types, improving the consistency and reliability of the validation process.

## Results

### Model Selection and Architecture

Different types of models, including CD-CNNs and pre-trained models, were evaluated. Supplementary Table 1 A presents the performance metrics for these models, tested on a dataset with a resolution of 50 × 50 pixels, 300 iterations, and a batch size of 32, which served as the baseline configuration. The models include both standalone versions and those combined with ELM. Metrics such as testing accuracy, specificity, sensitivity, and computational time (in minutes) are provided.

Among the models, model 3 + ELM stands out as the best-performing, achieving the highest testing accuracy (93.4%), specificity (97.1%), and sensitivity (88.0%). Its computational time was 7.43 min, which is relatively efficient compared to other models, especially larger ones like ResNet50 and DenseNet201, which required significantly more time. The second-best model, ResNet50, also demonstrated high testing accuracy, specificity, and sensitivity, albeit with longer computational times than model 3 + ELM. Supplementary Table 2 A provides results of model 3 + ELM evaluated across different batch sizes (8, 16, 32, 64, and 128). The model with a batch size of 32 performed the best.

### Classification Results

**Fig. 5:**
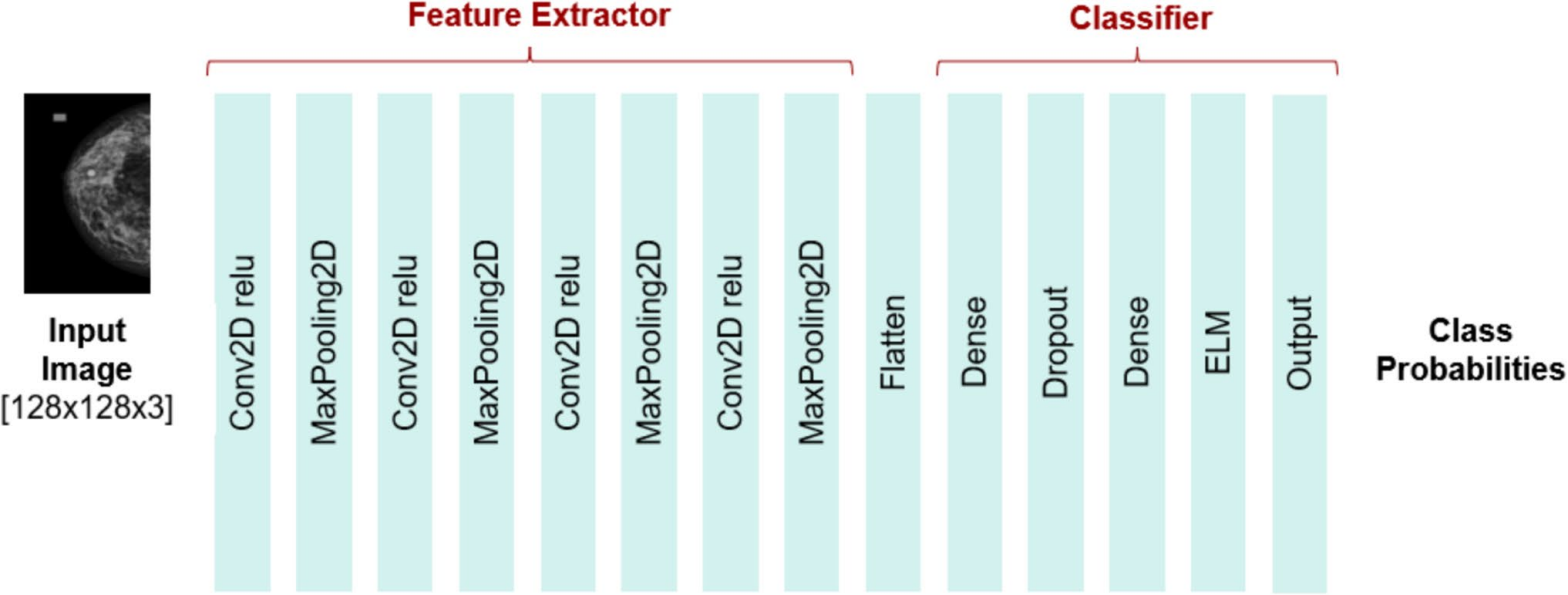
Simplified Architecture of model 3 + ELM

Figure 5 illustrates the simplified architecture of model 3 + ELM, which demonstrated the best performance and was therefore applied in this study. Input images were resized to 128 × 128 pixels and normalized to the 0–255 range. The model employed 10-fold cross-validation to evaluate the performance of a deep learning classifier based on a custom CNN architecture. This architecture included four convolutional layers, four max-pooling layers, three fully connected layers, and a dropout layer for regularization, along with custom layers and an extreme learning machine (ELM) layer.

The model was trained for 300 iterations, achieving an optimal balance between accuracy and computational efficiency, as detailed in Supplementary Table 3A. The configuration with 128 hidden units delivered the best performance using standard parameters. Employing 10-fold cross-validation with an input resolution of 128 × 128 pixels, the model achieved its highest overall performance, with a testing accuracy of 95.4%, specificity of 98.0%, sensitivity of 92.5%, and a computational time of 24.96 min, as reported in Supplementary Tables 4 A and 5A.

Table 2 presents the detailed performance metrics for model 3 + ELM based on 10-fold cross-validation conducted on the internal dataset. The model was evaluated for its ability to classify breast density into the four BI-RADS categories: A, B, C, and D.

**Table 2:** Performance metric results of Model 3+ELM, including results for each density category of BI-RADS (A, B, C, and D)

	**Accuracy**	**Specificity**	**Sensitivity**
	0.9540[0.9439–0.9641]	0.9801[0.9757–0.9848]	0.9251[0.8822–0.9474]
	**Precision**	**Recall**	**F1 Score**
**A**	0.9464[0.9324–0.9604]	0.9734[0.9601 - 0.9856]	0.9597[0.9518 - 0.9684]
**B**	0.9627[0.9339 - 0.9873]	0.9534[0.9384 - 0.9673]	0.9579[0.9465 - 0.9694]
**C**	0.9679[0.9163 - 1.0000]	0.8803[0.7430 - 0.9515]	0.9199[0.8487 - 0.9534]
**D**	0.9556[0.9130 - 0.9931]	0.8933[0.8437 - 0.9368]	0.9228[0.8975 - 0.9471]

The optimal model is configured with a batch size of 32, 300 iterations, and 128 hidden units in the Extreme Learning Machine (ELM), evaluated at a resolution of 128x128 using 10-fold cross-validation. Note: Statistically significant differences were found across BI-RADS classes for precision (ANOVA *p* = 0.0030), recall (*p* < 0.0001), and F1-score (*p* = 0.0001). According to Tukey’s HSD test, classes C and D had significantly lower values than classes A and B (*p* < 0.05), while no significant differences were observed between A vs B or C vs D.

The model demonstrated strong and consistent performance across all BI-RADS categories, achieving an overall accuracy of 95.4%, specificity of 98.0%, and sensitivity of 92.5%. BI-RADS A had the highest F1 score (96.0%), reflecting excellent precision and recall. BI-RADS B showed a precision of 96.3% and an F1 score of 96.8%. BI-RADS C achieved a precision of 96.8%, though its recall was slightly lower (88.0%), resulting in an F1 score of 92.0%. BI-RADS D exhibited high precision (95.6%) and a solid F1 score (92.3%).

Inter-rater agreement among specialists responsible for annotating mammograms, as well as between the specialists’ consensus and the density classification system, was assessed using a weighted kappa statistic. Results are presented in Table 3. Agreement between specialists was nearly perfect [25], with a kappa of 0.95 (95% CI: 0.89–1.00). In comparison, agreement between the density classification system and the specialists’ consensus demonstrated strong concordance, though slightly lower than the inter-specialist agreement, with a kappa of 0.90 (95% CI: 0.82–0.98).

**Table 3:** Inter-rater agreement between specialist 1 and specialist 2, as well as the agreement between the automated density classification system and specialist consensus, was assessed using the kappa coefficient and classification accuracy by category

**Kappa 0.95 (0.89, 1.00)**	**Specialist 2**					**Accuracy**
		**A**	**B**	**C**	**D**	
**Specialist 1**	**A**	5	2	0	0	71.4%
	**B**	0	5	0	0	100.0%
	**C**	0	0	13	0	100.0%
	**D**	0	0	1	4	80.0%
	**Total**	5	7	14	4	
**Kappa 0.90 (0.82, 0.98)**		**Breast Density AI Solution**				**Accuracy**
		**A**	**B**	**C**	**D**	
**Specialists’ Consensus**	**A**	7	0	0	0	100.0%
	**B**	1	4	0	0	80.0%
	**C**	0	4	7	2	53.8%
	**D**	0	0	0	5	100.0%
	**Total**	8	8	7	7	

Table 4 presents detailed information on the confusion matrix, showing how the model’s predictions align with actual classifications for the Mini-MIAS dataset. The overall accuracy of the model is 0.739 (95% CI: 0.692–0.787), reflecting the percentage of correct classifications across all categories. The total precision is 0.811 (95% CI: 0.769–0.853), indicating the proportion of predicted positives that are correctly classified. The total sensitivity, or recall, is 0.751 (95% CI: 0.704–0.798), measuring the model’s ability to correctly identify actual positives. The total specificity is 0.873 (95% CI: 0.837–0.909), demonstrating the model’s effectiveness in correctly identifying actual negatives.

**Table 4:** Confusion matrix for model predictions compared to actual classifications in the mini-MIAS dataset, categorized as fatty (F), fatty-glandular (G), and dense-glandular (D)

		**Breast Density AI Solution**		
		**F**	**G**	**D**
**Mini-MIAS**	**F**	99	9	1
	**G**	3	99	2
	**D**	5	66	46

## Discussion

The performance of pretrained models was not superior to that of model 3 + ELM, a CD-CNN, and they also required more computational time. Without the ELM layer, ResNet50 performed better than the other models. In most models, especially the pretrained ones, adding the ELM layer improved performance, supporting findings from other studies [26, 27]. The superior performance of model 3 + ELM can be attributed to its architecture, which is better suited to the unique characteristics of the dataset.

The results indicate that smaller batch sizes (8 and 16) increase computational time without significantly improving accuracy, specificity, or sensitivity compared to a batch size of 32. Larger batch sizes (64 and 128) reduce computational time but at the expense of performance metrics. Thus, a batch size of 32 offers the highest performance with reasonable computational time.

Increasing the number of iterations initially improves the model’s performance, but the benefits plateau and slightly decline beyond 300 iterations. Therefore, 300 iterations provide the optimal balance of performance metrics and computational efficiency. Similarly, increasing the number of hidden units beyond 128 does not lead to improved performance and may even degrade it. Thus, using 128 hidden units in the ELM layer is optimal, offering the best performance metrics and efficient computational time.

While increasing image resolution improves performance, it also significantly increases computational time. The model with tenfold cross-validation and a resolution of 128 × 128 pixels provides the best performance metrics. These results suggest that model 3 + ELM excels in identifying and classifying BI-RADS categories, with precision higher than 94.6% in all categories.

Most of the literature emphasizes identifying breast cancer rather than breast density [17, 18, 28–32], despite its recognized importance as a significant risk factor for breast cancer [5, 32, 33]. While some studies have achieved over 95.0% accuracy in breast density classification, there are notable variations in their methodologies. For instance, some studies classify mammograms into fewer than four categories [8, 34, 35], while others combine breast cancer information with breast density [12]. Additionally, several studies do not mention the use of cross-validation in their methodologies [9, 13, 16].

The system proposed in this research is on par with existing state-of-the-art methods, achieving an accuracy of 95.40% through cross-validation, ensuring greater confidence and generalization of the model. This system provides physicians with free access to a tool (https://breastdensity.streamlit.app/) that helps reduce the subjective nature of human observation and ambiguous results. Mammography classification based on density is subjective to specialists’ interpretations, identified as a major issue by experts in the field [8, 9].

The agreement between the automated density classification system and the specialists’ consensus is strong [25], with a weighted kappa statistic of 0.90 (95% CI: 0.82–0.98). This high kappa value suggests that the automated system generally aligns well with specialists’ ratings. However, the system faces greater challenges in accurately classifying category C, which slightly lowers its overall accuracy. These results highlight the high reliability among specialists who annotated the mammograms, with consistent classifications across cases. While the automated system shows strong agreement [25] with specialists, further refinements could enhance its ability to more distinctly classify category C, improving differentiation from categories B and D.

External validation using the Mini-MIAS dataset highlights the model’s strengths in precision and specificity. However, sensitivity shows room for improvement, as indicated by the weighted kappa results, especially for the dense-glandular category. The model could better differentiate between dense and transitional breast tissue types, suggesting an opportunity to enhance its discriminatory performance in these cases.

Several prior studies [8, 35–37] have evaluated breast density classification performance using the Mini-MIAS dataset, often reporting high accuracy—ranging from approximately 77 to 95%. However, a key methodological difference is that these studies predominantly relied on internal validation strategies, such as k-fold or leave-one-out cross-validation, where the same dataset (Mini-MIAS) was used for both training and testing. For instance, all trained and tested their models on Mini-MIAS using various cross-validation schemes. While such approaches are valuable for initial benchmarking, they may overestimate performance due to potential data leakage or overfitting to dataset-specific characteristics. In contrast, our study employed the Mini-MIAS dataset exclusively for external and independent validation, with no overlap with the training data. Despite this more stringent and realistic evaluation scenario, our model achieved an accuracy of 73.9%, precision of 81.1%, specificity of 87.3%, and sensitivity of 75.1%, which is competitive relative to prior work. This design provides a clearer estimate of the model’s generalizability to unseen data and demonstrates its potential applicability in real-world clinical settings, where performance on external data is critical. Consequently, while our metrics may appear modest compared to some internally validated results, they reflect a more rigorous validation protocol and offer stronger evidence of true model robustness.

These results illustrate that the open-source, publicly accessible nature of this automated classification system surpasses many commercial AI applications cited in the literature [10], which often involve significant financial barriers and may be unaffordable for public health systems. The system’s robust performance, free from commercial constraints, demonstrates the potential of open-source solutions to deliver reliable, cost-effective tools that can compete with proprietary options.

This study has several limitations that could be addressed in future research. First, it relied on BI-RADS breast density assessments conducted by interpretive radiologists in routine clinical settings. Additionally, two factors may limit the generalizability of the results: the external specialist validation relied on a small prospective dataset of 30 randomly selected mammography images, and external validation was performed exclusively using the Mammogram Image Analysis Society (MIAS) database, rather than multiple diverse datasets.

## Conclusion

This study presents a free and accessible approach to breast density assessment, delivering performance comparable to state-of-the-art methods. The system achieved an accuracy of 0.95 (95% CI: 0.94–0.96) and a weighted kappa coefficient of 0.90 (95% CI: 0.82–0.98). Implementing such solutions in public health systems, particularly in resource-limited settings, can optimize diagnostic workflows and significantly improve early breast cancer detection rates.

## Supplementary Information

ESM1 (DOCX 37.4 KB)

## Data Availability

The data supporting the findings of this study are available from the corresponding author upon reasonable request.
