# Supplementary material for: Enhancing Breast Density Assessment in Mammograms Through Artificial Intelligence
Source: J Imaging Inform Med. 2025 Sep 5;39(3):2188–98. doi: 10.1007/s10278-025-01657-6 (PMC13230292; doi:10.1007/s10278-025-01657-6)
Supplement: Supplementary file 1 — (DOCX 37.4 KB) [file 10278_2025_1657_MOESM1_ESM.docx]

**Supplemental Material**

| Model | Resolution | No. of iteration | Batch size | Testing accuracy | Specificity | Sensitivity | Computational time in minutes |
| --- | --- | --- | --- | --- | --- | --- | --- |
| MODEL1 | 50X50 | 300 | 32 | 0.9190 | 0.9650 | 0.8684 | 7.23 |
| MODEL1+ELM | 50X50 | 300 | 32 | 0.9224 | 0.9670 | 0.8587 | 7.27 |
| MODEL2 | 50X50 | 300 | 32 | 0.9243 | 0.9669 | 0.8636 | 10.52 |
| MODEL2+ELM | 50X50 | 300 | 32 | 0.9166 | 0.9625 | 0.8531 | 10.53 |
| MODEL3 | 50X50 | 300 | 32 | 0.9190 | 0.9655 | 0.8585 | 7.35 |
| MODEL3+ELM | **50X50** | **300** | **32** | **0.9344** | **0.9714** | **0.8805** | **7.43** |
| VGG16 | 50X50 | 300 | 32 | 0.9195 | 0.9651 | 0.8715 | 21.17 |
| VGG16+ELM | 50X50 | 300 | 32 | 0.9190 | 0.9648 | 0.8677 | 21.13 |
| ResNet50 | 50X50 | 300 | 32 | 0.9262 | 0.9693 | 0.8774 | 22.13 |
| ResNet50+ELM | 50X50 | 300 | 32 | 0.9243 | 0.9684 | 0.8757 | 22.22 |
| ResNet50V2 | 50X50 | 300 | 32 | 0.6920 | 0.8663 | 0.5263 | 21.36 |
| ResNet50V2+ELM | 50X50 | 300 | 32 | 0.6930 | 0.8597 | 0.4575 | 21.89 |
| VGG19 | 50X50 | 300 | 32 | 0.9065 | 0.9593 | 0.8559 | 25.78 |
| VGG19+ELM | 50X50 | 300 | 32 | 0.9118 | 0.9623 | 0.8636 | 25.99 |
| MobileNet | 50X50 | 300 | 32 | 0.6833 | 0.8573 | 0.4997 | 8.44 |
| MobileNet+ELM | 50X50 | 300 | 32 | 0.6891 | 0.8611 | 0.5022 | 8.43 |
| MobileNetV2 | 50X50 | 300 | 32 | 0.9016 | 0.9574 | 0.8505 | 11.50 |
| MobileNetV2+ELM | 50X50 | 300 | 32 | 0.9050 | 0.9583 | 0.8498 | 11.55 |
| DenseNet121 | 50X50 | 300 | 32 | 0.9079 | 0.9611 | 0.8515 | 21.57 |
| DenseNet121+ELM | 50X50 | 300 | 32 | 0.9074 | 0.9614 | 0.8556 | 20.94 |
| DenseNet201 | 50X50 | 300 | 32 | 0.9103 | 0.9613 | 0.8551 | 35.70 |
| DenseNet201+ELM | 50X50 | 300 | 32 | 0.9113 | 0.9625 | 0.8552 | 35.33 |

**Table 1A.** Performance measure results of models without cross-validation. The resolution is 50x50, with 300 iterations and a batch size of 32. Model 1 is a CNN with four convolutional layers, two fully connected layers, and four max-pooling layers. Model 2 is a CNN with eight convolutional layers, two fully connected layers, and four max-pooling layers. Model 3 is a CNN with four convolutional layers, three fully connected layers, and four max-pooling layers, additionally featuring a dropout layer for regularization. All pretrained models combined with Extreme Learning Machine (ELM) include custom fully connected layers (CFCL) consisting of flattening, dense layers with ReLU activation, and a 50% dropout rate.

| Model | Resolution | No. of iteration | Batch size | Testing accuracy | Specificity | Sensitivity | Computational time in minutes |
| --- | --- | --- | --- | --- | --- | --- | --- |
| MODEL3+ELM | 50X50 | 300 | 8 | 0.9219 | 0.9662 | 0.8719 | 24.55 |
| MODEL3+ELM | 50X50 | 300 | 16 | 0.9243 | 0.9674 | 0.8715 | 12.92 |
| MODEL3+ELM | **50X50** | **300** | **32** | **0.9344** | **0.9714** | **0.8805** | **7.43** |
| MODEL3+ELM | 50X50 | 300 | 64 | 0.9151 | 0.9635 | 0.8509 | 5.31 |
| MODEL3+ELM | 50X50 | 300 | 128 | 0.9228 | 0.9665 | 0.8748 | 4.15 |

**Table 2A.** Performance measure results of Model 3+ELM, a CNN with four convolutional layers, three fully connected layers, four max-pooling layers, and a dropout layer for regularization, without cross-validation. The resolution is 50x50, with 300 iterations, evaluated across different batch sizes (8, 16, 32, 64, and 128).

| Model | Resolution | No. of iteration | Batch size | Testing accuracy | Specificity | Sensitivity | Computational time in minutes |
| --- | --- | --- | --- | --- | --- | --- | --- |
| MODEL3+ELM | 50X50 | 100 | 32 | 0.9204 | 0.9659 | 0.8651 | 2.51 |
| MODEL3+ELM | 50X50 | 200 | 32 | 0.9243 | 0.9675 | 0.8801 | 4.94 |
| MODEL3+ELM | **50X50** | **300** | **32** | **0.9344** | **0.9714** | **0.8805** | **7.43** |
| MODEL3+ELM | 50X50 | 400 | 32 | 0.9224 | 0.9672 | 0.8660 | 9.98 |
| MODEL3+ELM | 50X50 | 500 | 32 | 0.9200 | 0.9661 | 0.8679 | 12.45 |

**Table 3A.** Performance measure results of Model 3+ELM, a CNN with four convolutional layers, three fully connected layers, four max-pooling layers, and a dropout layer for regularization, without cross-validation. The resolution is 50x50, with a batch size of 32, evaluated across different numbers of iterations (100, 200, 300, 400, and 500).

| Model | Hidden Units ELM | Resolution | No. of iteration | Batch size | Testing accuracy | Specificity | Sensitivity | Computational time in minutes |
| --- | --- | --- | --- | --- | --- | --- | --- | --- |
| MODEL3+ELM | 64 | 50X50 | 300 | 32 | 0.8775 | 0.9491 | 0.8233 | 7.50 |
| MODEL3+ELM | **128** | **50X50** | **300** | **32** | **0.9344** | **0.9714** | **0.8805** | **7.43** |
| MODEL3+ELM | 256 | 50X50 | 300 | 32 | 0.8838 | 0.9519 | 0.8352 | 7.56 |
| MODEL3+ELM | 512 | 50X50 | 300 | 32 | 0.8737 | 0.9464 | 0.8146 | 7.65 |

**Table 4A.** Performance measure results of Model 3+ELM, a CNN with four convolutional layers, three fully connected layers, four max-pooling layers, and a dropout layer for regularization, without cross-validation. The resolution is 50x50, with a batch size of 32 and 300 iterations, evaluated across different numbers of hidden units in the Extreme Learning Machine (ELM) layer (64, 128, 256, 512). The size of the last fully connected layer in the CNN is adjusted to match the number of hidden units in the ELM layer, ensuring consistent input dimensions and simplifying integration.

| Model | k-fold cross validation | Hidden Units ELM | Resolution | No. of iteration | Batch size | Testing accuracy | Specificity | Sensitivity | Computational time in minutes |
| --- | --- | --- | --- | --- | --- | --- | --- | --- | --- |
| MODEL3+ELM | 5 | 128 | 50X50 | 300 | 32 | 0.8932 | 0.9556 | 0.8475 | 7.65 |
| MODEL3+ELM | 5 | 128 | 128X128 | 300 | 32 | 0.9487 | 0.9778 | 0.9214 | 23.29 |
| MODEL3+ELM | 5 | 128 | 224X224 | 300 | 32 | 0.8881 | 0.9535 | 0.8375 | 63.75 |
| MODEL3+ELM | 10 | 128 | 50X50 | 300 | 32 | 0.9010 | 0.9585 | 0.8564 | 7.93 |
| MODEL3+ELM | **10** | **128** | **128X128** | **300** | **32** | **0.9540** | **0.9801** | **0.9251** | **24.96** |
| MODEL3+ELM | 10 | 128 | 224X224 | 300 | 32 | 0.9516 | 0.9791 | 0.9241 | 68.12 |

**Table 5A.** Performance measure results of Model 3+ELM, a CNN with four convolutional layers, three fully connected layers, four max-pooling layers, and a dropout layer for regularization. The model is evaluated with a batch size of 32, 300 iterations, and 128 hidden units in the Extreme Learning Machine (ELM) across different resolutions (50x50, 128x128, and 224x224) and different k-fold cross-validation settings (k=5 and k=10).
